# Modelling biological systems from molecules to dynamical networks

**DOI:** 10.1186/1752-0509-6-S1-S1

**Published:** 2012-07-16

**Authors:** Yong Wang, Xiang-Sun Zhang, Luonan Chen

**Affiliations:** 1 Academy of Mathematics and Systems Science, Chinese Academy of Sciences, Beijing 100190, China; 2 Key Laboratory of Systems Biology, SIBS-Novo Nordisk Translational Research Centre for PreDiabetes, Shanghai Institutes for Biological Sciences, Chinese Academy of Sciences, Shanghai 200031, China

## Abstract

A report of the 5^th ^IEEE International Conference on Systems Biology (IEEE ISB2011), 2-4 September 2011, Zhuhai, China.

## Background

It's almost 10 years since the computational systems biology concept has been proposed in 2002 [1]. We witnessed the impact of collaboration and integration among biology, physics, mathematics and computer science on biological and medical research in the past decade. As a result, this energetic interdisciplinary field, i.e., computational systems biology keeps making significant progresses to address fundamental questions in biology and to further lead to practical applications in medicine, drug discovery, and bio-engineering. We are glad to see that the expanding trend is still there with the boom of next generation sequencing technology.

In addition to the progress on individual research works, the past decade also witnessed the boom of societies of computational systems biology. In China, Systems Biology and Bioinformatics have become intensive research topics and attracted many leading scientists working in biology, physics, mathematics, and computer science. Optimization, statistics, and many other mathematical methods have been widely adopted in the field. Four years ago, we launched an international symposium on Optimization and Systems Biology, which focused on bridging opportunities between optimization methodology and systems biology problems. We are happy to see the mutual benefits both in solving specific biological problems and in new theory and algorithm development [2]. In 2010, OSB conference was formally renamed as International Conference on Computational Systems Biology (ISB2010) [3]. The successful International Conferences on Optimization and Systems Biology (OSB2007, OSB2008, and OSB2009) and the International Conference on Computational Systems Biology (ISB2010) have created a flexible platform and brought many researchers and students to freely exchange ideas [2,3]. However a conference is not an organization and is limited in its power to continuously support the development of the interdisciplinary field. It is in pressing need to extend the conference to a well-organized research society for scientists, researchers, educators, and practitioners to exchange ideas and approaches, to present research findings, find state-of-the-art solutions, and organize various activities in this interdisciplinary field, including mathematical methods and its applications in biosciences and researches on a variety of aspects of Systems Biology.

Therefore, Prof. Xiang-Sun Zhang, the honorary president of Operations Research Society of China (ORSC), proposed to establish a computational systems biology society under ORSC to emphasize the integration of experimental and computational research to understand the complex biological systems. The idea was greatly supported by Prof. Ya-Xiang Yuan (the president of ORSC) and also the scientists in this field. As a result, the Computational Systems Biology Society of ORSC in China was formally set up in 2011 (website http://www.sysbio-cn.org/). Initially the society has over 120 members and a council with 35 board members representing the universities and research institutes all over China. The board member elected Prof. Luonan Chen, a professor from Shanghai Institutes for Biological Sciences, to be the founding president of this new society. Importantly, the society will keep supporting the series conference of ISB and has made it the annual conference for all the members. In addition, the Technical Committee on Systems Biology of IEEE Systems, Man, and Cyberstics society decided to financially sponsor the 5th International Symposium on Computational Systems Biology (ISB 2011), with the goal to provide a forum for exchanging ideas and information among researchers and engineers in the field of Systems Biology from engineering perspectives.

As a result, this conference is formally renamed as the 5th IEEE International Conference on Computational Systems Biology (IEEE ISB2010). To match the name, we call for the participants from an even wider range. In addition to the sponsorships from IEEE SMC, Computational Systems Biology Society of ORSC, National Natural Science Foundation of China (NSFC), Academy of Mathematics and Systems Sciences of CAS (AMSS), Shanghai Institutes for Biological Sciences of CAS (SIBS), it was further sponsored by Sun Yat-Sen University.

We strongly believe that the joint efforts of societies, funding agencies, research institutes, and universities will further push the development of computational methodologies for systems biology and the practical applications in biology, medicine, drug discovery, and engineering in future.

## Meeting report

A three-day international conference on Computational and Systems Biology was held in 2-4 September, 2011 (IEEE ISB2011) in Zhuhai, which is a beautiful city in south China. More than 100 researchers including engineers, physicians, mathematicians, and biologists from China mainland, United States, Hong Kong, Taiwan, Japan, Korea, Australia, and Singapore enjoyed both academic exchanges and natural scenes. In the opening session of IEEE ISB2011, Prof. Luonan Chen announced and all the participants celebrated the establishment of Computational Systems Biology Society in ORSC of China.

The Proceedings of the 5th International Conference on Computational Systems Biology (IEEE ISB2011) have been published by IEEE and are available online http://ieeexplore.ieee.org/xpl/mostRecentIssue.jsp?punumber=6026104. Sixty-three papers in this volume cover wide range of computational systems biology and all the papers are indexed by EI. Moreover, the reviewers from the Program Committee of IEEE ISB2011 selected 21 papers to be recommended for a special issue in BMC Systems Biology after significant extension of their original versions on the Proceedings. Each submission has been peer reviewed and evaluated by three independent reviewers on the quality, originality, soundness, and significance of its contributions and the significant improvement regarding to the IEEE ISB2011 proceeding paper. Here we focus on some of the highlights of the meeting by categorizing and briefly introducing these selected papers.

## Network systems biology

One of the most important keyword for computational systems biology is network [4,5]. Biomolecular network concept is proposed to understand cellular behavior in the systems level in terms of the spatiotemporal interactions among cellular components, such as genes, proteins, metabolites, and organelles. It has emerged as a powerful approach to study complex biological processes and created new words such as "network biology" and "network medicine". In this issue, Guanying Piao et al. reconstructed the active regulatory networks involved in diabetes progression in Goto-Kakizaki rats and developed a computational procedure for identifying biologically plausible transcriptional master regulators. Shu-Qiang Wang and Han-Xiong Li proposed a binding affinity based regulatory model to quantify the transcriptional regulatory network. Jing Zhao et al. applied a network based systems approach for the identification of candidate genes related to Axial spondyloarthropathy, which is a group of chronic inflammatory joint diseases that mainly affect the spine and the sacroiliac joints. Kejia Xu et al. constructed a 'drug cocktail network' using all the known effective drug combinations extracted from the Drug Combination Database, and proposed a network-based approach to investigate drug combinations. Xiaoke Ma and Lin Gao developed a novel core-attachment based method for protein complexes detection in protein interaction network by transforming the original problem into a classic all-clique problem. Chen Chen et al. noticed that biological network exhibits general properties of complex networks, such as `scale-free' and `small-world' and integrated this prior information into the parsimony-based model to accurately reconstruct protein-protein interaction network. Xi Chen et al. studied the computational method to find the optimal control policy for Probabilistic Boolean Network, which is a popular model for studying genetic regulatory networks. All these works demonstrate the fact that network is a powerful approach in the post genome era by studying genome-wide networks and it has far reaching implication in general complex network research [6].

## Dynamics systems biology

Another keyword for systems biology is dynamics [7]. Dynamics is an important phenomenon at systems level and it is inherent within the biological systems because proper cellular functioning requires the precise coordination of a large number of events [8]. In this issue, Canjun Wang et al. studied the combined effects of the correlated noise and time delays on the gene regulatory model. Shuyun Jiao and Ping Ao started from the point of the adaptive landscape to illustrate the dynamical behaviors for Muller's ratchet, which describes an important process that the accumulation of deleterious mutations of a population directly contributes to the fate as to how long the population would exist. Ru-Dong Li and Lei Liu developed a method to directly assess protein system-level properties based on system dynamics and in silico knockouts, regarding to the conceptual term "criticality" and found that multiple enzymes including phosphoglycerate kinase, enolase, transketolase-b, etc., had critical roles in the system in terms of both system states and dynamical stability. Tao Zeng and Luonan Chen proposed a causal process model to systematically study the biological phase transition, which widely exists in the biological world, such as transformation of cell cycle phases, cell differentiation stages, disease development, and so on. Wei Zhang and Xiufen Zou investigated and modeled a multi-cell system of the Xenopus embryonic cell cycle oscillators that are coupled through a common complex protein, and then analyzed their synchronization ability under four different external stimuli, including a constant input signal, a square-wave periodic signal, a sinusoidal signal and a noise signal. These works show that dynamics concept can be used to widely study the nonlinear mechanisms of complex biological phenomena.

## High throughput data analysis and integration

In addition to network and dynamics, the most important aspect of systems biology is data analysis and integration [9]. Particularly computational systems biology predicates on the integration of experimental data from an ever increasing number of technologies, such as gene expression arrays, proteomics, and chromatin immunoprecipitation on chip assays, to deliver useful information about the system of interest. In this issue, Xianfu Gao et al. performed a parallel metabolomics study, based on human urine and serum, to comprehensively profile systematic metabolic variations, identify differential metabolites, and understand the pathogenic mechanism of membranous nephropathy. Yong Wang et al. generated the high-throughput metabolic profiles of acupuncture treatment at several acupoints in human and developed a novel Linear Programming based Feature Selection method to understand the mechanism of acupuncture effect, at molecular level, by revealing the metabolite biomarkers for acupuncture treatment. Xiaoquan Su et al. proposed Parallel-META, a GPU- and multi-core-CPU-based open-source pipeline for metagenomic data analysis, which enabled the efficient and parallel analysis of multiple metagenomic datasets and the visualization of the results for multiple samples. Chao Dai et al. modelled each RNA-seq dataset as a co-splicing network, where the nodes represent exons and the edges are weighted by the correlations between exon inclusion rate profiles, designed a tensor-based approach to identify co-splicing clusters that appear frequently across multiple conditions, and applied their method to the 38 co-splicing networks derived from human RNA-seq datasets to indentify an atlas of frequent co-splicing clusters. These studies demonstrated the wide coverage of computational systems biology studies by analyzing the data from proteomics, metabolomics data to next generation sequencing data, such as RNA-seq and metagenomics data.

## Molecular systems biology

In addition to computational study at the systems level, computational systems biology approaches also can be utilized to study the molecular function of single gene or protein, because cellular functions of genes involved in specific biological processes depend on genetic, physical, and other types of interactions. In this issue, Min Xu and Frank Alber proposed a gradient-guided alignment methods based on two subtomogram similarity measures, a real space as well as a Fourier-space constrained score to align and average a large set of cryo-electron subtomogram, which emerges as an important component for structural system biology. Hari Krishna Yalamanchili et al. designed a novel automated protein functional assignment method based on the neural response algorithm, which simulates the neuronal behavior of the visual cortex in the human brain, to fill the gap between the number of sequences and their annotations. Yan Zhang et al. conducted comparative genomics studies to identify a novel DNA-binding protein, YebC, which may serve as a key transcriptional regulator that mainly regulates the gene expression of RuvABC resolvasome in bacteria.

## Evolutionary systems biology

From a more general perspective, evolutionary systems biology focuses on one of the core problems of all biology, i.e., the evolutionary interplay between the genotype and the phenotype [10]. The bidirectional gene architecture has been studied in many organisms, and the conservation of bidirectional arrangement has received considerable attention. In this issue, Chao Xu et al. identified the bidirectional gene pairs in eight different species and found this structure to be prevalent in eukaryotes. It is well known that identifying corresponding genes (orthologs) in different species is an important step in genome-wide comparative analysis. Melvin Zhang and Hon Wai Leong presented a simple yet effective method (BBH-LS) for the identification of positional homologs from the comparative analysis of two genomes and integrated sequence similarity and gene context similarity in order to get more accurate ortholog assignments.

## Competing interests

The authors declare that they have no competing interests.

## Authors' contributions

YW drafted the manuscript. XSZ and LC read and approved the manuscript.
